# The Emerging Role(s) for Kisspeptin in Metabolism in Mammals

**DOI:** 10.3389/fendo.2018.00184

**Published:** 2018-04-24

**Authors:** Andrew Wolfe, Mehboob A. Hussain

**Affiliations:** ^1^Department of Pediatrics, Johns Hopkins University, Baltimore, MD, United States; ^2^Department of Internal Medicine Metabolism, Endocrinology and Diabetes, University of Michigan, Ann Arbor, United States

**Keywords:** kisspeptin, obesity, pancreas, liver, adipocytes, mouse models

## Abstract

Kisspeptin was initially identified as a metastasis suppressor. Shortly after the initial discovery, a key physiologic role for kisspeptin emerged in the regulation of fertility, with kisspeptin acting as a neurotransmitter *via* the kisspeptin receptor, its cognate receptor, to regulate hypothalamic GnRH neurons, thereby affecting pituitary–gonadal function. Recent work has demonstrated a more expansive role for kisspeptin signaling in a variety of organ systems. Kisspeptin has been revealed as a significant player in regulating glucose homeostasis, feeding behavior, body composition as well as cardiac function. The direct impact of kisspeptin on peripheral metabolic tissues has only recently been recognized. Here, we review the emerging endocrine role of kisspeptin in regulating metabolic function. Controversies and current limitations in the field as well as areas of future studies toward kisspeptin’s diverse array of functions will be highlighted.

## Introduction

### Historical Summary of Kisspeptin

The kisspeptin (*Kiss1*) gene was first identified in a screen of human genes that reduced the metastatic potential of human melanoma cells (1). Since its discovery, kisspeptin has been a focus of study for a series of different fields including cancer biology, reproductive neuroendocrinology, reproductive biology, and, most recently, metabolism. While the kisspeptin gene and its activation of the kisspeptin receptor (KISS1R) were initially characterized by cancer biologists (1, 2), in 2003, the study of kisspeptin accelerated following the demonstration by two groups of its essential role in regulating reproductive function (3, 4). This can be dramatically illustrated by performing a literature search for articles on kisspeptin from the years between 1996 and 2002 (18 articles) and the subsequent 7 years, 2003–2009 (458 articles), after the seminal studies from Seminara and de Roux. The studies from this latter period define a critical role for kisspeptin signaling in the regulation of GnRH neurons, demonstrating kisspeptin involvement with puberty (3, 4), mediating gonadal steroid hormone negative (5, 6) and positive (7, 8) feedback and serving as an afferent pathway for metabolic control of the reproductive hormone axis (9–11). Interest in kisspeptin has further accelerated in the past 7 years (1,540 articles) as novel peripheral roles for kisspeptin have been identified in both reproductive, metabolic, and developmental processes (12–14). The aim of this review is to provide a summary of studies describing a role for kisspeptin in the peripheral regulation of metabolism.

### Kisspeptin and the KISS1R

**Figure 1:**
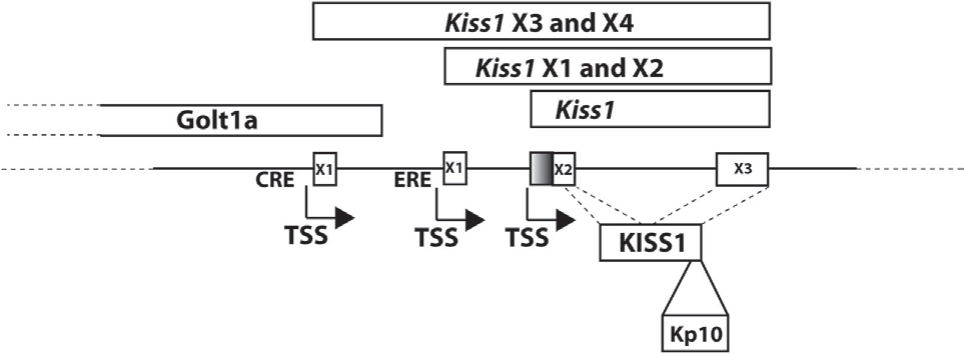
Mouse *Kiss1* gene. Transcript variants of mouse *Kiss1* are expressed from two different first exons, X3 (XM_006529681) and X4 (XM_006529682), that are regulated by cyclic AMP response element binding protein at a CRE (19). The transcriptional start site for *Kiss1X*3 and X4 is located in an exon of the Golt1a gene. *Kiss1X*1(XM_006529679) and X2 (XM_006529680) are regulated by ESR1 at an ERE (17). *Kiss1* is a transcript including just the second and third exons (NM_178260.3). X1 and X3 include a larger second exon (including the shaded region) and X2 and X4 include a smaller second exon. All variants produce the KISS1 protein. KP-10 is the active region of all bioactive KISS1 peptides.

The kisspeptin gene, located on chromosome 1 in human, was originally reported to encode a 145 amino acid preprotein (15), though recent updates to the human genome sequence include a one-bp change resulting in an earlier stop codon and indicating that the human protein product is likely 138 amino acids. The preprotein can be further processed to the biologically active 54 amino acid C-terminally amidated peptide (Kp54, metastin) that was demonstrated to activate the KISS1R previously referred to as the orphan receptor GPR54 (2, 16). In mouse, the *Kiss1* gene, as in human, is located on chromosome 1, and in rat, the gene is located on chromosome 13. However, for the rat and mouse genes, regulation is complicated by the expression of multiple splice variants, although, in both, the protein precursor is also processed to Kp54. For example, the rodent *Kiss1* gene (mouse gene shown in Figure 1) consists of a number of splice variants that produce the same protein product (17, 18), suggesting that key differences in cell-specific regulation may be mediated by alternative promoter elements. This has been borne out in studies which have defined cyclic AMP response element binding protein (CREB) (19) and estrogen receptor (17) regulation of the mouse *Kiss1* gene (Figure 1).

The *Kiss1* gene is expressed in a variety of tissues besides the brain (hypothalamus, amygdala) (20–23), including the liver (13, 24), testis (24–26), ovary (27, 28), fetal adrenal (12), heart (29) fat (24, 30, 31), and placenta (32). This provides a number of possibilities for sources of circulating kisspeptin, with strong experimental evidence suggesting that the liver and placenta can contribute to biologically significant levels in the circulation (13, 32).

The KISS1R gene (*Kiss1r*, also called *Gpr54, Axor12*, and hOT7T175) is a member of the G-protein-coupled receptor family and is located on chromosome 19 in human and chromosome 10 in mouse. It consists of five exons and encodes a 398 amino acid protein in humans and a 395 amino acid protein in the mouse (33).

The Kiss1R has significant homology in the transmembrane regions with the galanin receptors, yet has little affinity for galanin (34). Kiss1R was demonstrated to signal through a G_q/11_-mediated pathway to increase intracellular Ca^2+^ (35, 36) and activate the extracellular signal-regulated kinase (ERK)-signaling pathway, stimulating GnRH secretion. Recently, in the GnRH neuron, KISS1R was also demonstrated to signal *via* a G_q/11_-independent but β-arrestin-dependent pathway leading to the activation of ERK (37, 38).

Besides the hypothalamus (39, 40), *Kiss1r* has been reported to be expressed in peripheral tissues including the pituitary, adipocyte, heart, spinal cord, gonads, and pancreas (13, 16, 29–31, 41–43). These findings suggest that a range of physiological systems may be impacted by kisspeptin.

Though not activated by galanin, the Kiss1R has been demonstrated to be activated by ligands other than the kisspeptins. RFamide-related peptide-3 (RFRP-3) and its receptor, neuropeptide FF receptor 1 (NPFFR1), are the mammalian orthologs of avian gonadotrope inhibitory hormone (GnIH) and its receptor GnIHR. RF9 is an antagonist to the GnIHR (44) that directly activates GnRH neuron firing (45) and LH secretion in a KISS1R-dependent manner (46). While kisspeptin independent activation of the KISS1R is documented, high levels of kisspeptin can also exert effects independent of the KISS1R (13) though the mechanism of action for these effects is not yet established. These data likely provide a biological rationale for the more severe reproductive phenotype observed in the *Kiss1R* KO mouse than in the *Kiss1* KO mouse (47).

### Kisspeptin Neurons Mediate Central Regulation of Reproduction by Peripheral Metabolic Signals

Since the initial observations describing an essential role for kisspeptin signaling in puberty (3, 4), it has emerged that kisspeptin neurons are also relays of steroid feedback regulation to GnRH neurons (5, 6, 21, 22, 39, 48, 49) and are important components of the circuitry controlling GnRH pulse generation (50–54).

Kisspeptin neurons in the brain have also been proposed to integrate signals relaying peripheral metabolic status to the reproductive hormone axis, specifically to the GnRH neurons [reviewed in Ref. (9, 55, 56)]. There is evidence for changes in central kisspeptin expression both in response to food restriction (negative energy balance) or in genetic or diet-induced models of obesity (positive energy balance). However, to date, reports on the modulation of kisspeptin expression by caloric surfeit and obesity vary and are at times conflicting such that no firm consensus exists on the topic.

In studies on calorically restricted models, most, but not all, investigators report a reduction in *Kiss1* expression in both hypothalamic kisspeptin neuron populations. Long-term diet-restricted ewes were shown to express reduced *Kiss1* mRNA in both the ARC and POA when compared with normal weight ewes (57). And in fasted male mice, reduced hypothalamic *Kiss1* mRNA levels relative to fed controls were reported (58). In rats, one group reported 72 h of fasting caused a significant reduction in hypothalamic *Kiss1* expression in both males and females (59). However, another group observed no change in ARC *Kiss1* expression in response to a 48-h fast and a reduction in AVPV *Kiss1* mRNA only in ovariectomized/estrogen-replaced female rats (60). Clearly, more studies will be required to address the role of hypothalamic kisspeptin in mediating the suppression of the reproductive axis in states of negative energy balance.

In diet-induced obese female mice, *Kiss1* expression in both the AVPV and ARC decreases relative to normal chow-fed controls (61), perhaps contributing to a reduced reproductive function. And in a genetic model of obesity, the leptin-deficient Ob/Ob mouse, ARC *Kiss1* mRNA levels are reduced (49, 61, 62) or unchanged (58) as compared to control mice. In the study by Smith et al., leptin treatment of Ob/Ob mice increased *Kiss1* expression, but this represented only a partial rescue of a reduced *Kiss1* expression reported for the Ob/Ob mouse (21, 22, 49). Leptin has long been known to play a permissive role in reproductive function (63) and signaling *via* the kisspeptin neurons, which may contribute to the functional regulation of fertility by leptin.

The gut-derived hormone ghrelin could also impact the reproductive axis *via* kisspeptin neuronal afferents, though here again, the evidence is not clear. Some investigators have suggested that only the AVPV KISS1 neurons are regulated by ghrelin (64), and others have also identified the regulation of ARC KISS1 neurons by ghrelin, interestingly with a strong interacting effect by E2 (65). Kisspeptin neurons have been shown to express the ghrelin receptor [GHSR (65)], though they are not thought to express the leptin receptor (62, 66), suggesting leptin-sensitive afferent neuronal regulation of kisspeptin and/or GnRH neurons (62, 66). ARC kisspeptin neurons send projections to the kisspeptin neurons in the AVPV (67, 68), suggesting that for some processes, a two kisspeptin neuron circuit could be required. Therefore, the relative role that ARC or AVPV kisspeptin neurons play in mediating metabolic signals is not clear (60, 61, 64, 69, 70).

### The *Kiss1R* Knockout Mouse Exhibits Metabolic Dysfunction and Suggests a Key Role for Kisspeptin Signaling in Regulating Metabolism

Evidence for a broader role for kisspeptin signaling in the regulation of metabolism comes from detailed analysis of the KISS1R knockout mouse (*Kiss1r* KO) (71). These studies reported striking differences in body weight and glucose metabolism in female mice, but also differences in body composition and increased circulating leptin in both sexes. Because of the well-established obesity associated with a reduced estrogen signaling (72–74), Tolson et al. ovariectomized the female mice to assess those effects occurring independently of reduced estrogen. They found that a small, but significant, component of the obesity observed in female mice is due to loss of kisspeptin signaling and is not secondary to the reduction in estrogen levels, resulting from hypothalamic hypogonadism (71). However, *Kiss1r* KO males, because they exhibited no KISS1R-mediated weight difference, were not as carefully studied, despite having an increased adiposity and circulating leptin compared to controls. In addition, not reported in either sex was any evaluation of gluconeogenic capacity or whether there were differences in lipid metabolism despite an observed reduction in nocturnal respiratory exchange ratio (RER) in female KO mice compared to controls as assessed by indirect calorimetry. A reduced RER is suggestive of an increased use of lipids for energy metabolism (75). In follow-up studies in female mice, the Kauffman laboratory demonstrated that the changes in body composition, leptin levels, and RER were present in 6-week-old female KO mice, which preceded the increased body weight phenotype (76). The developmental progression of the phenotype observed in the male KO mice (increased adiposity, leptin levels, and reduced RER) remains still to be performed. The studies by the Kauffman laboratory have spurred a number of investigators to try to assess the tissue-specific mechanisms by which kisspeptin may regulate glucose and lipid metabolism, food intake, and body weight.

One possibility is that the body weight phenotype in Kiss1r KO mice is in part the result of an altered hypothalamic control of food intake or energy expenditure. Kisspeptin treatment alters both neuropeptide Y (NPY) and pro-opiomelanocortin (POMC) neuronal activity in mice (77). In sheep, kisspeptin treatment increases *Npy* gene expression and decreases *Pomc* expression (57). Using optogenetic technology, ARC kisspeptin neurons were activated in mice and demonstrated glutamine, secreted from kisspeptin neurons regulation of both POMC and agouti-related peptide neurons. Interestingly, this resulted in the excitation of POMC neurons, *via* Gq/G11 coupled glutamate receptor and the inhibition of AgRP neurons, *via* a G_i_/G_o_ coupled glutamate receptor (78).

To address whether the underlying cause of the obesity in female *Kiss1r* KO mice was at the level of the hypothalamus, De Bond et al. used quantitative PCR and *in situ* hybridization histochemistry to examine the expression of key genes in the hypothalamic appetite-regulating system, including *Pomc* and *Npy* as well as the genes expressing the receptors for leptin, ghrelin, and the melanocortins (79). They established that there were no differences in the expression of any of these genes between ovariectomized *Kiss1r* KO and ovariectomized control mice, suggesting peripheral sites of action of kisspeptin as potentially being a primary contributor to altered metabolism in the *Kiss1r* KO mouse. However, these sorts of assessments of mRNA levels do not preclude possible changes in neuronal activity and/or neurotransmitter release. They also do not fully address heterogeneity of the relevant neuronal populations and suggest that additional studies are needed to fully clarify a possible central role for kisspeptin signaling in energy metabolism. The following sections address the potential role of kisspeptin signaling in the major metabolic organs in the body, specifically the liver, muscle, fat, and pancreas.

### Liver-Derived Kisspeptin Participates in Islet Hormone Cross-Talk

A long-standing question in pancreatic islet biology is how glucagon, produced in α-cells in response to low glucose levels, influences insulin secretion from β-cells that occurs in states of hyperglycemia. These considerations have clinical relevance since patients with type 1 diabetes mellitus (T1DM) exhibit hyperglucagonemia (80). In addition, many patients with type 2 diabetes mellitus (T2DM) exhibit elevated glucagon levels and insufficient insulin secretion to control glucose levels (81, 82). High glucagon levels are also apparent in prediabetic patients, who exhibit impaired glucose tolerance, suggesting that impaired glucagon suppression may contribute to the development of T2DM (83, 84).

The glucagon receptor is expressed on hepatocytes, where its activation rapidly stimulates cyclic AMP (cAMP) production (85), activating the PKA-signaling cascade. The PKA regulatory complex consists of two catalytic subunits (C) and two regulatory subunits (R). The C subunits are sequestered by the R subunits in the absence of cAMP. Increases in cAMP result in the release of the C subunits which phosphorylate and activate CREB and increase the transcription of CREB-responsive genes. These include genes for the rate-limiting enzymes for gluconeogenesis, such as glucose-6-phosphatase (*G6pase*) or phosphoenolpyruvate carboxykinase (*Pepck*) (86–88), providing the adaptive response of an increased hepatic glucose production in response to hypoglycemia.

Constitutive activation of liver PKA-dependent signaling stimulates gluconeogenesis, leading to hyperglycemia, which would be expected to stimulate insulin secretion from β-cells. However, experimentally, the opposite has been observed. In 2005, the McKnight laboratory developed a mouse model with liver-specific expression of a mutant PKA C subunit (tryptophan 196 to arginine, called CαR) that exhibits impaired binding to the PKA R subunit and is thus less sequestered (inactive) in the absence of cAMP. The CαR mice did not have an increased expression of the gluconeogenic enzymes *G6pase* or *Pepck*, but did have reduced hepatic glycogen levels and were found to have modest hyperglycemia, but this was not associated with higher insulin levels, but rather with a reduced insulin secretion (89). Similar results were observed by our group using a more robust model of constitutive hepatic PKA activation, one in which the PKA R subunit gene is completely disrupted by Cre/LoxP technology (L-ΔPrkar1a mice). These mice have an increase in *G6pase* and *Pepck* gene expression in the liver causing fasting hyperglycemia and, notably, insufficient insulin secretion to correct glycemia during intraperitoneal glucose tolerance tests (13). These data suggest that an increased PKA signaling in the liver could be indirectly acting on pancreatic β-cells to suppress insulin secretion.

Evidence that a secreted factor was mediating this effect came from a bioassay in which plasma from L-Prkar1a mice suppressed insulin secretion from wild-type (WT) mouse islets *in vitro* (13).

To identify the factor, we compared hepatic gene expression in the L-ΔPrkar1a mouse with WT mice infused with glucose to achieve hyperglycemia equivalent to that in the L-ΔPrkar1a mouse. Of note, glucose-infused WT mice exhibited a robust and significantly elevated insulin secretion in contrast to the L-ΔPrkar1a counterparts. A liver gene expression array combined with bioinformatic analysis to identify genes for secreted proteins that were upregulated in the liver of L-ΔPrkar1a mice surprisingly yielded a single candidate gene, *Kiss1*, that was significantly upregulated in L-ΔPrkar1a liver (13). This result was confirmed by direct assessment of liver kisspeptin mRNA expression by quantitative PCR as well as kisspeptin protein levels by immunoblot.

Glucagon is secreted during fasting to participate in adaptive energy mobilization in the liver and fat. We demonstrated that *Kiss1* expression was increased in overnight fasted WT mice, but not in mice with a liver-specific deletion of the glucagon receptor gene. These results suggest that liver glucagon receptor activation can both stimulate insulin secretion by increasing blood glucose levels and inhibit insulin secretion by stimulating kisspeptin production.

To confirm the functional regulation of insulin secretion by the kisspeptin receptor, we used mice with selective ablation of the pancreatic *Kiss1r* gene (Panc-Kiss1R mouse) using the pancreas-specific PDX-1 CRE driver mouse and a *Kiss1r* floxed mouse that we developed (40). Acute treatment of control mice with kisspeptin preceding a glucose injection resulted in impaired glucose tolerance and attenuated insulin secretion, while Panc-Kiss1R mice injected with kisspeptin before glucose injection had glucose tolerance and insulin secretion similar to vehicle-injected mice (13).

Attempts to assess a role for kisspeptin on insulin secretion have yielded conflicting results with some noting that kisspeptin stimulates glucose-stimulated insulin secretion (GSIS) (90, 91) and others reporting the opposite (92, 93). We noted a wide range of concentrations for kisspeptin used in these various studies, with kisspeptin concentrations in the nM range usually suppressing GSIS (92, 93) and μM kisspeptin concentrations usually stimulating GSIS (90, 91, 94). To directly address this controversy, we tested different concentrations of kisspeptin on GSIS in islets from control and Panc-Kiss1R mice. We found that kisspeptin at nM concentrations suppressed GSIS from control islets but not from islets lacking the KISS1R. By contrast, kisspeptin at μM concentrations stimulated GSIS even in the absence of the KISS1R (Panc-Kiss1R islets). Based on these studies, it is clear that the suppression of GSIS by nanomolar concentrations of kisspeptin is mediated by the KISS1R. At supraphysiological levels, kisspeptin stimulates GSIS through a non-KISS1R-mediated pathway.

We assessed liver expression of kisspeptin in mouse models of obesity. Both high-fat diet (HFD) fed obese and genetic models of obesity (db/db and Ob/Ob mice) had an increased liver kisspeptin expression as well as increased circulating plasma kisspeptin concentrations (13). To assess whether these results translated to humans, liver biopsies taken from patients diagnosed with T2DM were analyzed and exhibited a higher kisspeptin expression than liver tissue from non-diabetic subjects. This was associated with higher circulating kisspeptin levels in diabetic subjects than in non-diabetic subjects (13). These findings suggest that in T2DM, kisspeptin production is elevated in the liver and that this increased kisspeptin production is secondary to increased glucagon levels, and, indeed, treatment with a glucagon receptor antagonist in db/db mice reduced liver kisspeptin production and improved glucose homeostasis (13).

**Figure 2:**
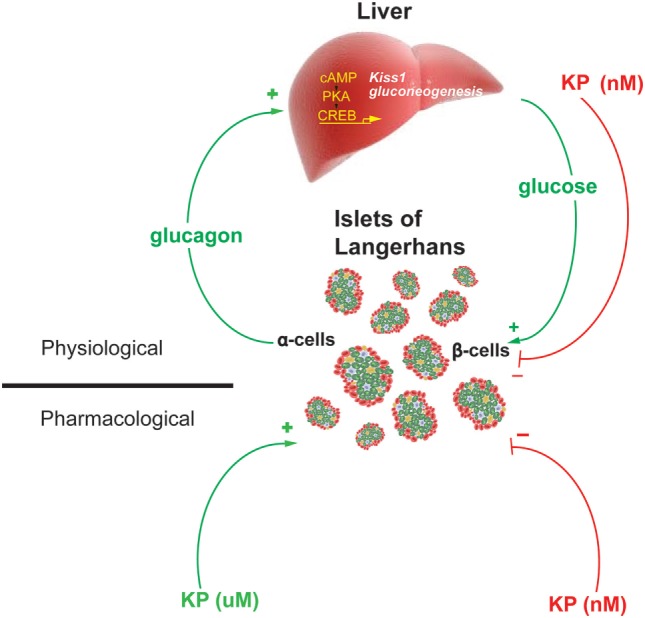
Model of the proposed Hepato-Islet circuit. Glucagon secreted from the pancreatic α-cell activates its receptor on the liver increasing PKA activity and gene expression of gluconeogenic genes and the *Kiss1* gene. Increased glucose output from the liver increases blood glucose levels, increasing insulin secretion from the pancreatic β-cells. Increased kisspeptin secretion from the liver acts to suppress glucose-stimulated insulin secretion from the pancreatic β-cells. Pharmacologically delivered kisspeptin can stimulate (μM levels) or inhibit (nM levels) GSIS. Figure adapted from Song et al. (13).

Therefore, these data demonstrate the existence of a hepatopancreatic circuit in which glucagon, from the pancreas, stimulates hepatic expression of the genes regulating gluconeogenesis and kisspeptin. While the increased expression of *Pepck* and *G6pase* increases hepatic glucose output, increases blood glucose levels and stimulates insulin secretion, the increased secretion of kisspeptin serves to suppress insulin secretion (Figure 2). Kisspeptin could therefore be developed as a therapeutic in the treatment of some metabolic disease.

### Placenta Is a Major Source of Circulating Kisspeptin in Humans

Our data suggest that the liver contributes to circulating levels of kisspeptin that in metabolically challenged states can increase 2- to 10-fold above basal (13); however, these levels are far lower than those secreted in women at the end of pregnancy by the placenta [elevated nearly 10,000-fold (32)]. This dramatic increase has been corroborated in a recent study in which urine kisspeptin levels were over 200-fold higher in third trimester pregnant women than in non-pregnant women (95). Kisspeptin levels decline rapidly after delivery, supporting the placenta as the source (32). Human term placenta was analyzed for *Kiss1* mRNA by *in situ* hybridization and for kisspeptin by immunohistochemistry, and kisspeptin expression in the outer syncytiotrophoblasts was reported, ideally located for secreting kisspeptin into the maternal circulation (32). The authors noted that there are similarities between invasive placental cells and invasive cancer cells (32), and during the establishment of the maternal–fetal interface, it is important to limit the interaction between the trophoblasts and uterine cells. Perhaps, kisspeptin plays a role in this process, mirroring its originally defined role as a metastasis inhibitor (1). Therefore, the increase in the production of kisspeptin in syncytiotrophoblasts in the first trimester may also play a role in negatively regulating trophoblast invasion, and kisspeptin signaling might be required for implantation and placentation (96).

A metabolic role for placentally derived kisspeptin can also be envisioned. During normal human pregnancy, insulin resistance develops and peaks during late pregnancy [34–36 weeks of gestation (97)]. This may be an adaptive response to preserve a slight excess of energy substrates in the blood for use by the developing fetus. A number of hormones have been proposed to contribute to the development of insulin resistance, including human placental lactogen, human placental growth hormone, progesterone, cortisol, tumor necrosis factor α, and leptin (97). One could envision two possible explanations for the high kisspeptin levels late in pregnancy. If kisspeptin is acting *via* the KISS1R, it would serve to tamp down increased insulin secretion to maintain a modest excess in the blood levels of energy substrates (glucose, free fatty acids). Intriguingly, insulin sensitivity recovers very quickly after delivery in parallel with decreasing kisspeptin levels (32, 97). One could also propose that the very high levels of kisspeptin circulating late in pregnancy could act *via* a kisspeptin receptor independent mechanism and serve to augment insulin secretion to compensate for the increased insulin resistance in late pregnancy.

Further investigation of placental kisspeptin will be difficult using many animal models. Very modest levels of placental kisspeptin expression have been reported in the rat, dog, and mouse (98–100). And while a gestational increase in kisspeptin expression was noted for mouse (99) and dog (98), circulating levels of kisspeptin are unlikely to reach the levels observed in humans and may not play the same role in these animal models as compared to humans.

### Fat May Also Be a Source of Circulating Kisspeptin

The adipocyte could also be a source of circulating kisspeptin. *Kiss1* mRNA has been detected in rat adipose tissue (30, 31), and food restriction increased Kiss1 mRNA in the fat of both male and female rats (30). T1DM but not T2DM was associated with roughly 100-fold higher *Kiss1* mRNA levels in adipocytes compared to non-diabetic rats (24), suggesting that insulin plays a key role in the regulation of adipocyte *Kiss1* expression. Interestingly, the large increase in *Kiss1* mRNA was not associated with increased kisspeptin protein levels in the adipocytes of T1DM rats, suggesting either increased secretion or reduced protein translation. By contrast, Kiss1 mRNA was reduced in obese HFD fed and obese Zucker rats (30). The Wilkerson group also demonstrated a sex steroid regulation of adipocyte *Kiss1* expression. Estradiol stimulated expression in female adipocytes and testosterone stimulated expression in male adipocytes (30).

The adipocyte also expresses the KISS1R (101). Therefore, kisspeptins secreted by adipose tissue could either act as adipokines or as autocrine/paracrine regulators of adipocyte function. To explore kisspeptin’s effects in fat, 3T3-L1 and primary rat hepatocytes were treated with Kp10 and lipid metabolism, glucose uptake and leptin and adiponectin secretion assessed (101). These studies demonstrated that Kp10 reduced adipogenesis in 3T3-L1 cells, likely as a result of a reduced expression of peroxisome proliferator-activated receptor gamma (PPAR-γ) and CCAAT/enhancer binding protein beta (CEBPβ), transcription factors involved in stimulating adipogenesis. Kp10 increased lipolysis in 3T3-L1 cells and rat adipocytes by enhancing the expression of periliphin and hormone-sensitive lipase and decreased glucose uptake and lipogenesis. Kp10 also stimulated the secretion of leptin and decreased the secretion of adiponectin from rat adipocytes. While these studies suggest a role for kisspeptin in regulating adipocyte development and function, the effects were largely seen at near μM levels of Kp10, calling into question the physiological relevance of the findings. It is possible that local levels of paracrine/autocrine secretion of kisspeptin could reach these levels, or that the very high levels of kisspeptin observed during human pregnancy could achieve levels that functionally regulate fat, although this is unlikely to play a role in mouse or rat given the relatively modest levels of kisspeptin during gestation in these rodent models.

Human fat has also been demonstrated to express *Kiss1* (31). In women, a positive correlation between *Kiss1* mRNA in visceral adipose tissue and body mass index (BMI) was reported (31). Exclusions for this study included women under 19 years old and those that were post-menopausal. Not excluded were subjects with diabetes. These findings agree with our observation that circulating kisspeptin levels are increased 2- to 4-fold in HFD fed and db/db obese mice and nearly 10-fold in humans with T2DM when compared to lean mice and non-diabetic humans, respectively (13). These findings are at odds with the rodent data showing a reduced *Kiss1* expression in obese rats (30), and they also appear to differ from studies showing decreased circulating kisspeptin levels in obese patients with BMIs above 35 kg/m^2^ when compared to non-obese controls with BMIs below 25 kg/m^2^ (102). However, the high BMI subjects in the Kolodziejskii study specifically excluded those with diabetes and they would not exhibit hyperglucagonemia and the resulting increased hepatic *Kiss1* expression (102).

The contribution of fat to circulating levels of kisspeptin is unclear, making it difficult to discern whether kisspeptin from fat serves as an endocrine factor. A cell-specific KO of the kisspeptin gene from adipocytes would help address this question. These studies may ultimately demonstrate an exclusively paracrine/autocrine role of kisspeptin in fat regulation.

### Other Potential Effects of Kisspeptin on Peripheral Metabolic Function

Evidence for the muscle as a target or a source of kisspeptin is limited. While skeletal muscle has not been demonstrated to synthesize kisspeptin or express significant levels of the KISS1R, there is evidence that smooth and cardiac muscles are regulated by kisspeptin.

Kisspeptin receptor has been localized in cardiomyocytes as well as the smooth muscle cells of the intramyocardial blood vessels (29, 103), and kisspeptin has been demonstrated to induce inotropic actions on cardiac function with the effects confirmed to be mediated by the KISS1R (29). The relevance during normal physiology is unclear, however, since no cardiac dysfunction is reported in either humans or mice lacking the KISS1R (29). It was proposed that the high levels of kisspeptin secreted from the placenta could play a role in the adaptive increase in cardiac output during pregnancy (29, 104). However, the local expression of kisspeptin-like immunoreactivity was noted in human, mouse, and rat vascular and endocardial endothelial cells and in human cardiomyocytes (29) and could also be a source of high kisspeptin levels. Local secretion of kisspeptin could be a mechanism for intracardial regulation of cardiac output. Interestingly, the level of kisspeptin immunoreactivity was significantly lower in the right atria of patients transplanted for ischemic heart disease when compared to controls. While these changes could be secondary to low oxygen levels, it could suggest a possible role for kisspeptin in maintaining proper blood flow to the heart during atherosclerotic arterial narrowing.

Regulation of gut motility also contributes to metabolic status. A recent report suggests that kisspeptin can stimulate gastrointestinal motility by both central and peripheral mechanisms (105). While ICV infusion of kisspeptin stimulated gastrointestinal motility and fecal output at low nM concentrations, kisspeptin also exerted direct effects on the contractility of the circular smooth muscle of the colon. However, the peripheral effects of kisspeptin in the colon were only apparent at μM concentrations and could indicate a non-KISS1R-mediated mechanism of action such as observed for the effects of high kisspeptin concentrations on the beta cell (13). Local secretion of kisspeptin could achieve μM levels and represent an endogenous regulatory mechanism in the gastrointestinal system. Alternatively, these studies may help define a therapeutic role for pharmacological kisspeptin.

Kisspeptin does not appear to directly impact energy metabolism of skeletal muscle, but the literature does indicate a potentially important role on cardiac function and gut motility. Leveraging conditional knockout mouse models of both kisspeptin and the KISS1R will be required to fully understand kisspeptin’s role in regulating heart contractility and gut motility.

### Summary and Conclusion

**Figure 3:**
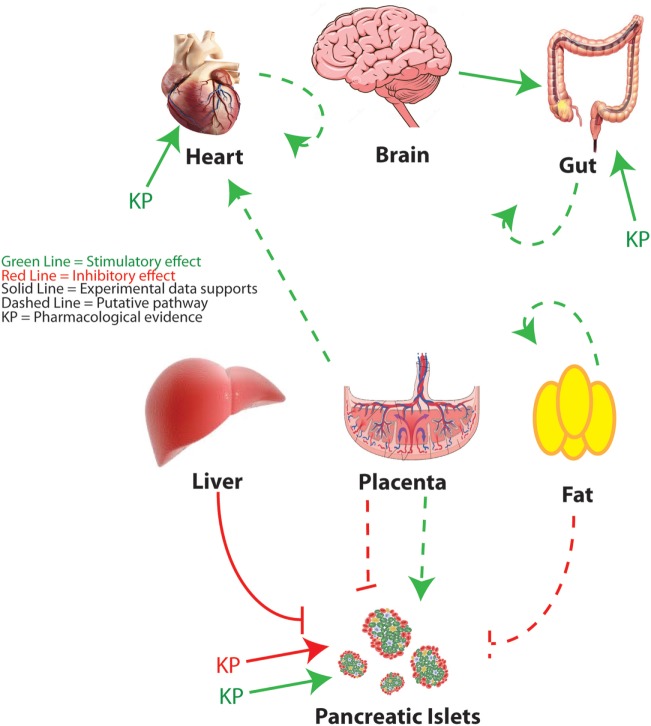
Peripheral metabolic regulation by kisspeptin. An overview of kisspeptin regulatory pathways is discussed in this review. Red lines indicate putative suppressive effects. Green lines indicate putative stimulatory effects. Solid lines indicate that specific experimental evidence is provided to support the pathway. Dashed lines indicate a speculative pathway based on the available evidence. KP indicates targets for which pharmacological roles for kisspeptin have been proposed. Figure of brain adapted from Dreamstime.com.

As the study of kisspeptin enters its third decade, and new functions are attributed to the peptide, more animal and human studies are needed to understand its complex pleiotropic effects. The widespread expression of kisspeptin and its receptor indicates an ever-expanding array of roles in normal physiology, but also during the extreme physiological, developmental, and metabolic challenges of pregnancy or in pathophysiological states such as diabetes (Figure 3). In reviewing the literature, several challenges emerge. The first is that a spectrum of kisspeptin doses is being used, both *in vivo* and *in vitro*, and more attention needs to be paid to whether the effects of kisspeptin are physiological or pharmacological. This is not meant to disparage the latter since there is evidence that kisspeptins show a therapeutic potential in a variety of systems. A second challenge is trying to understand whether kisspeptins’ effects are being mediated by the kisspeptin receptor and, if not, to determine those mechanisms of action not mediated by the cognate receptor. When possible, it is invaluable to validate the mechanism of action using the Kiss1R KO mouse (13, 29). The development of novel mouse models, including mice with floxed alleles of both the kisspeptin (in development) and KISS1R (40, 106) genes and optogenetic tools to assess neurobiological circuitries (50, 51, 107), will help define the sources of kisspeptin and the relevant sites of action. However, the development of additional animal and human models will be imperative to adequately study phenomenon not recapitulated in rodent models.

## Author Contributions

AW and MH jointly conceived of and wrote the manuscript.

## Conflict of Interest Statement

The authors declare that the research was conducted in the absence of any commercial or financial relationships that could be construed as a potential conflict of interest.
